# Turbulent transport reduction induced by transition on radial electric field shear and curvature through amplitude and cross-phase in torus plasma

**DOI:** 10.1038/s41598-017-14821-y

**Published:** 2017-11-02

**Authors:** T. Kobayashi, K. Itoh, T. Ido, K. Kamiya, S.-I. Itoh, Y. Miura, Y. Nagashima, A. Fujisawa, S. Inagaki, K. Ida

**Affiliations:** 1 National Institute for Fusion Science, National Institutes of Natural Sciences, Toki, 509-5292 Japan; 2 Institute of Science and Technology Research, Chubu University, Kasugai, 487-8501 Japan; 3 Research Center for Plasma Turbulence, Kyushu University, Kasuga, 816-8580 Japan; 4 National Institute for Quantum and Radiological Science and Technology, Naka, 311-0193 Japan; 5 Research Institute for Applied Mechanics, Kyushu University, Kasuga, 816-8580 Japan; 6 Japan Atomic Energy Agency, Tokai, 319-1184 Japan

## Abstract

Spatiotemporal evolutions of radial electric field and turbulence are measured simultaneously in the H-mode transition, which is a prototypical example of turbulence structure formation in high-temperature plasmas. In the dynamical phase where transport barrier is established abruptly, the time-space-frequency-resolved turbulent particle flux is obtained. Here we report the validation of the mechanism of transport barrier formation quantitatively. It is found that the particle flux is suppressed predominantly by reducing density fluctuation amplitude and cross phase between density fluctuation and potential fluctuation. Both radial electric field shear and curvature are responsible for the amplitude suppression as was predicted by theory. Turbulence amplitude reduction immediately responds to the growth of the radial electric field non-uniformity and saturates, while cross phase continuously approaches zero.

## Introduction

Structure of flows and turbulence in non-equilibrium plasmas has attracted much attention because of its great impact on the entire media dynamics. One of the prototypical examples can be seen in the solar tachocline^1^, across which the transition from the differential rotation in the solar convective zone to the rigid rotation in the radiative interior occurs. The strong shear flow within the thin layer is believed to amplify the magnetic field as the solar dynamo. Another example is the edge transport barrier (ETB) in toroidal fusion plasmas^2^, which is spontaneously formed in the Low-confinement mode to High-confinement mode transition (L-H transition)^3^. Because of its rich nature of nonlinear dynamics as well as its promising capability for achieving good plasma performance toward the fusion reactor, much attention has been paid for clarifying the underlying physics for decades. Numerous studies have shown essential roles of an edge-localized poloidal *E* × *B* flow structure on confinement improvement^4–7^. However, definitive conclusion regarding what aspect of the *E* × *B* flow suppresses the turbulent transport is still under debate^8^. In order to obtain an understanding based on first principles, interplay between the flow structure and the turbulence must be diagnosed with high spatiotemporal resolutions, which remains challenging.

In modeling works, roles of inhomogeneous *E* × *B* flow on the turbulence suppression are classified in two elements: shear and curvature. Effects of both non-uniformities on mode instability were discussed^9,10^. Nonlinear saturation level of turbulence was studied using a statistical approach, showing an essential role of both shear and curvature of the *E* × *B* flow^11^. In a newly developed model^12^ described in “Method”, shear and curvature were simultaneously treated in a single formulae that provided turbulence reduction rate. In that model, responsible physics of shear and curvature were considered as the *E* × *B* flow shear decorrelation of turbulence eddy^13–15^ and the modulational coupling causing energy transfer from turbulence to macroscopic *E* × *B* flow structure^2,12,16^, respectively. Experimental examinations of those models have been performed^8,17–25^. Focusing on detailed physics of transport suppression, direct fluctuation measurement by use of electrostatic probes has been promoted. It was found that the turbulent particle flux is reduced not only by the density fluctuation amplitude suppression but also by the cross phase alternation between the density fluctuation and the potential fluctuation^19–22^. Although individual elements regarding the turbulent transport suppression by the inhomogeneous *E* × *B* flow have been raised, i.e., shear and curvature of *E* × *B* flow and amplitude and cross phase of fluctuations, the mutual relation of these elements remain unclear.

In this paper, we investigate response of turbulent particle flux on radial electric field non-uniformity by analyzing data from a heavy ion beam probe (HIBP). In particular, interrelations among shear and curvature of radial electric field and amplitude and cross phase of fluctuations are shown for the first time. Electron density and electrostatic potential measured with high spatiotemporal resolutions allow us to perform time-space-frequency-decomposition to the fluctuation induced particle flux. The particle flux is suppressed predominantly by reducing both the density fluctuation amplitude and the cross phase between density fluctuation and potential fluctuation. Both curvature and shear are responsible for transport reduction. Turbulence amplitude reduction immediately responds to the growth of the radial electric field non-uniformity and saturates, while cross phase continuously approaches zero. The time scales of these dynamics have an order of magnitude difference. As a result, turbulent transport reduction occurs with two different time scales.

## Results

### Time-space-frequency-resolved turbulence spectrum

**Figure 1:**
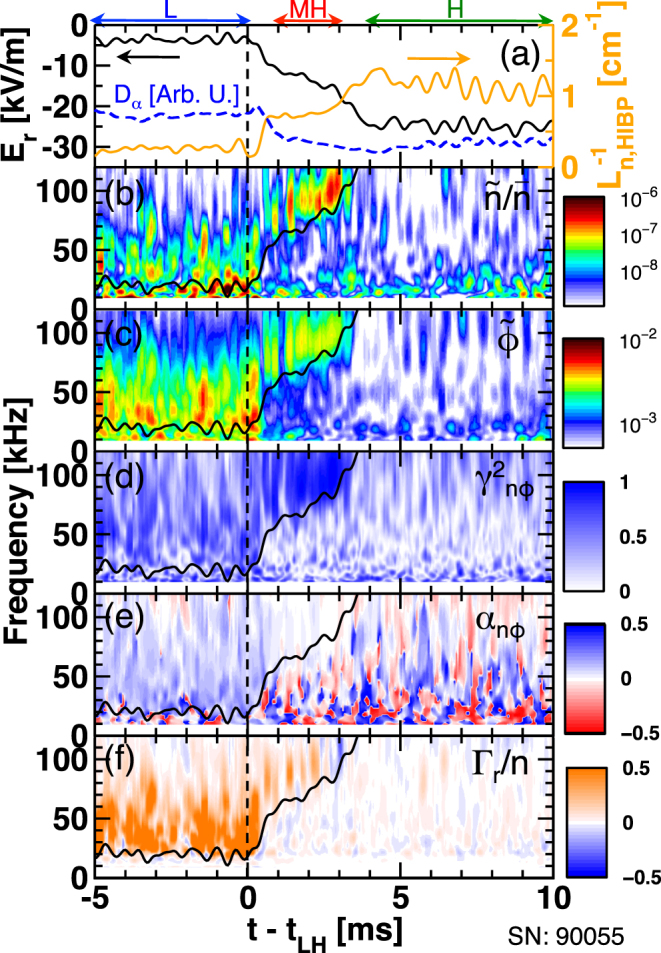
Time traces of (**a**) ${D}_{\alpha }$ emission, inverse density gradient length and radial electric field, wavelet power spectra of (**b**) relative density fluctuation and (**c**) potential fluctuation, (**d**) and (**e**) squared cross coherence and cross phase between density fluctuation and potential fluctuation, respectively, and (**f**) particle flux normalized by mean density at $r-a\sim -0.8$ cm. Black curves on contour plots show the expected frequency Doppler shift in the laboratory frame.

Figure 1 (a) shows time evolutions of ${D}_{\alpha }$ emission from divertor region, inverse density gradient length ${L}_{n,{\rm{HIBP}}}^{-1}\equiv -\nabla {I}_{{\rm{HIBP}}}/{I}_{{\rm{HIBP}}}$, where ${I}_{{\rm{HIBP}}}$ is the HIBP secondary beam current that reflects the local electron density, and radial electric field ${E}_{r}$ at $r-a\sim -0.8$ cm, in which the bottom of the *E*_*r*_-well structure emerges in H-mode. The global profile changes are represented by low-pass filtered signals with a cut-off frequency of 2 kHz. The cut-off frequency is chosen to eliminate dynamics of the limit-cycle oscillation at 4.5 kHz^26^. As clearly indicated, transition in ${L}_{n,{\rm{HIBP}}}^{-1}$ and *E*_*r*_ occurs twice^27^. The first transition is triggered by the reach of a sawtooth crash-induced heat pulse at the edge region. The onset time ${t}_{{\rm{LH}}}$ is defined using the ${D}_{\alpha }$ emission signal and a soft-x-ray signal. After the first transition within several hundred microseconds, the plasma experiences a short period quasi-stationary state of ∼2 ms, which we call the “MH-mode” (meta-stable H-mode). Then, the plasma reaches the final H-mode by completing the transport barrier with the second transition within ∼1 ms. A dedicated study revealed that the radial current induced by the neoclassical bulk viscosity^4,5^, and the ion loss-cone loss^5^ plays an important role for building *E*_*r*_ structure during the first transition^28^. Reynolds stress driven *E*_*r*_ was found to be much smaller.

Contour plots in Fig. 1 show time-frequency-resolved wavelet power spectra of (b) relative density fluctuation $\tilde{n}/\bar{n}$ and of (c) potential fluctuation $\tilde{\varphi }$, (d) and (e) squared cross coherence and cross phase between $\tilde{n}$ and $\tilde{\varphi }$ denoted as ${\gamma }_{n\varphi }^{2}$ and ${\alpha }_{n\varphi }$, respectively, and (f) particle flux normalized by mean density ${\Gamma }_{r}/n$. Here, wavelet transform is performed with a time interval of 50 *μ*s, after which four sequential time samples are ensemble averaged. Squared cross coherence and cross phase are defined as ${\gamma }_{n\varphi }^{2}=|{P}_{n,\varphi }{|}^{2}/{P}_{n}{P}_{\varphi }$ and ${\alpha }_{n\varphi }={tan}^{-1}{\rm{Re}}[{P}_{n,\varphi }]/\mathrm{Im}[{P}_{n,\varphi }]$ with the power spectrum of $\tilde{n}$ and $\tilde{\varphi }$ and cross spectrum between them denoted as ${P}_{n}$, ${P}_{\varphi }$, and ${P}_{n,\varphi }$, respectively. The particle flux is defined as ${{\rm{\Gamma }}}_{r}={B}^{-1}{({P}_{n}{P}_{\varphi }{\gamma }_{n\varphi }^{2})}^{1/2}{k}_{\theta }\sin {\alpha }_{n\varphi }$ ^29^. The poloidal wavenumber of turbulence *k*_*θ*_ is determined to be ${k}_{\theta }\sim \,0.75\,{{\rm{cm}}}^{-1}$ in $f > 20$ kHz in a dedicated discharge, in which the sample volumes of HIBP are aligned in a magnetic surface at $r-a\sim -1$ cm^30^. The poloidal wavenumber in $f < 20$ kHz is much smaller compared to that in $f > 20$ kHz so that the particle flux driven by those low frequency components play a minor role for confinement. In this paper, the poloidal direction is defined as the electron diamagnetic direction. At the central frequency $f\sim 50$ kHz, phase velocity is approximately equal to the electron diamagnetic velocity in the plasma frame.

Properties of turbulence and turbulent particle flux change drastically through the transitions. In L-mode, a broadband turbulence spectrum in $f < 80$ kHz arises both in $\tilde{n}/\bar{n}$ and $\tilde{\varphi }$ spectra. Cross phase ${\alpha }_{n\varphi }$ is slightly positive in average and turbulence-driven particle flux is directed outward. During the L-MH transition, the central frequency of the turbulence spectrum rises sharply up to ∼100 kHz. Black curves superimposed on Fig. 1 (b)-(f) show the expected Doppler frequency shift for turbulence component at $f=20$ kHz in L-mode, where ${k}_{\theta }$ is assumed to remain unchanged through L-MH transition. Below the curve, amplitude of fluctuation is significantly reduced, in which fluctuation wavenumber is found to be substantially lower than that of the high frequency turbulence component^30^. In contrast, the higher frequency component suffers a moderate amplitude reduction. Squared cross coherence ${\gamma }_{n\varphi }^{2}$ remains close to unity but cross phase ${\alpha }_{n\varphi }$ decreases toward zero. A considerable reduction of the particle flux occurs, which is responsible for density pedestal formation. Further deepening of the *E*_*r*_-well occurs in the final H-mode. Doppler shifted frequency exceeds measurable frequency band. Therefore we avoid investigating the final H-mode period in this paper.

**Figure 2:**
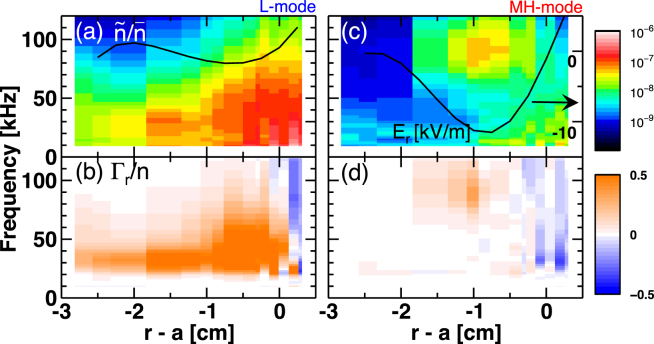
Radial profiles of time averaged wavelet spectra of (**a** and **c**) relative density fluctuation and (**b** and **d**) particle flux normalized by mean density in L-mode and in MH-mode, respectively. Black curves in (**a**) and (**c**) are radial profile of radial electric field.

Figure 2 shows radial profile of time-averaged turbulence spectrum. Radial profile of *E*_*r*_ is superimposed as black curves. In L-mode, turbulence has a broad spectrum both in frequency and in space. This broadband turbulence generates a widely distributed outward directed particle flux that is related to the confinement degradation. After the L-MH transition, the *E*_*r*_-well structure with the full width at half maximum of ∼1.4 cm emerges at $r-a=-0.8$ cm. In MH-mode, Doppler frequency shift occurs up to ∼100 kHz with a moderate amplitude reduction at the bottom of the *E*_*r*_-well. The Doppler frequency shift is only visible within the *E*_*r*_-well. While, the turbulence is strongly stabilized in the outer shear region ($r-a > -0.3$ cm), the inner shear region ($-1.8\,{\rm{cm}} < r-a < -1.3$ cm), and further inside ($r-a < -1.8\,{\rm{cm}}$). As a result, the outward particle flux is entirely suppressed outside the *E*_*r*_-well. Even inside the *E*_*r*_-well, the particle flux is clearly reduced mainly because of change in the cross phase ${\alpha }_{n\phi }$ as discussed above.

### Response of turbulent particle flux on radial electric field non-uniformity

**Figure 3:**
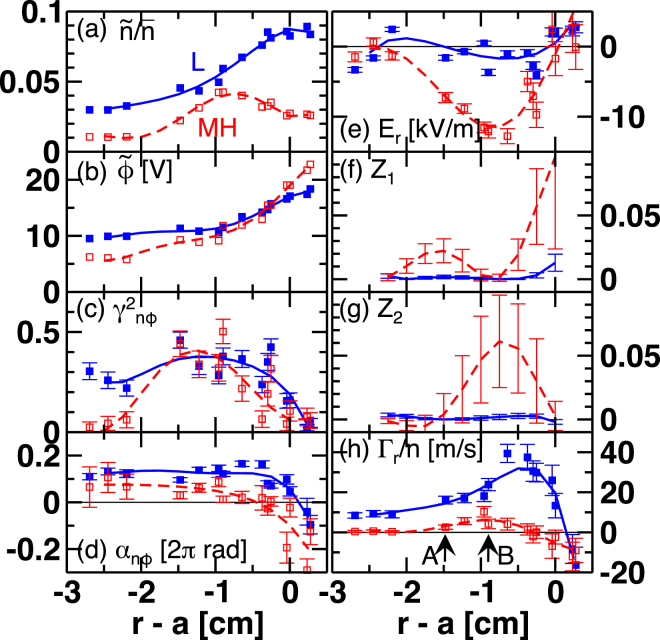
Radial profiles of (**a**) relative density fluctuation amplitude, (**b**) potential fluctuation amplitude, (**c**) and (**d**) squared cross coherence and cross phase between density fluctuation and potential fluctuation, respectively, (**e**) radial electric field, (**f**) shear parameter ${Z}_{1}$, (**g**) curvature parameter ${Z}_{2}$, and (**h**) particle flux normalized by mean density. Arrows labeled “A” and “B” in (**h**) indicate radii in which detailed time traces are shown in Fig. 4.

On frequency integrated turbulence properties shown in Fig. 3, we quantitatively show response of turbulent particle flux on radial electric field non-uniformity. Radial profiles of fluctuation amplitude for $\tilde{n}/\bar{n}$ and $\tilde{\varphi }$ [Fig. 3(a) and (b)] are given by integrating the spectra in $20\,{\rm{k}}{\rm{H}}{\rm{z}}\le f\le 110\,{\rm{k}}{\rm{H}}{\rm{z}}$. In L-mode, shapes of both amplitude profiles are similar and normalized turbulence amplitudes are approximately in the same level, $e\mathop{\varphi }\limits^{ \sim }/{T}_{{\rm{e}}} \sim \mathop{n}\limits^{ \sim }/\bar{n}$, where an equivalent order of electron temperature and ion temperature ${T}_{{\rm{e}}}\sim {T}_{{\rm{i}}}$ is assumed^7^, and ${T}_{{\rm{i}}}\sim 130$ eV is obtained by a charge exchange spectroscopy. A small but finite phase difference between density fluctuation and potential fluctuation exists. The dominant turbulence source is considered as the resistive drift wave^26^. Two different elements of radial electric field non-uniformity, shear and curvature, are parameterized as non-dimensional factors *Z*_1_ and *Z*_2_, respectively. See “Method” for the definition. Radial profiles of *Z*_1_ and *Z*_2_ are shown in Fig. 3(f) and (g). Spatial derivative is taken for fifth order polynomial fit of the *E*_*r*_ profile. The model^12^ predicts that the non-uniformity observed here is large enough to suppress turbulence amplitude, i.e., ${(k{\rho }_{{\rm{i}}})}^{-2}({Z}_{1}+{Z}_{2}) > 1$, where the turbulence scale factor is given as ${(k{\rho }_{{\rm{i}}})}^{-2}\sim 0.01$. Note that the criterion for ${Z}_{1}$ is equivalent to the well-known shearing rate criterion^13^, i.e., $\partial |{E}_{r}/B|/\partial r > {\gamma }_{{\rm{L}}}$, if the turbulence decorrelation rate ${\gamma }_{{\rm{L}}}$ can be approximated by the drift wave frequency $k{V}_{{\rm{d}}}$. The criterion for *Z*_2_ corresponds to $\sqrt{|{E}_{r}{E}_{r}^{^{\prime\prime} }/{B}^{2}|} > k{V}_{{\rm{d}}}$ ^16^. At the location where ${(k{\rho }_{{\rm{i}}})}^{-2}{Z}_{2} > 1$ is held, a substantial fraction of the microscopic turbulence energy is transferred into the macroscopic radial electric field. If the transferred energy is large enough to maintain the radial electric field structure, the H-mode transition can be described by an integrated system of physics, which is referred to as “single-step” transition in Ref.^31^. In the preset case, however, the turbulence driven radial electric field accounts for only a small fraction of the total one^28^ (referred to as “two-step” transition^31^).

Comparing the $\tilde{n}/\bar{n}$ profiles in L-mode and MH-mode, *Z*_1_ seems to be more effective for reducing turbulence amplitude than *Z*_2_. Note that around the radius of the *Z*_2_ peak, the density pedestal appears in which linear energy input to the turbulence is enhanced. The $\tilde{\varphi }$ profile has a pivot point at $r-a\sim -0.4$ cm, inside or outside which the turbulence amplitude decreases or increases, respectively. A sign dependence of *E*_*r*_ shear for $\tilde{\varphi }$ suppression possibly exists as discussed in Ref.^19^. A mild reduction in ${\gamma }_{n\varphi }^{2}$ is also seen. Cross phase ${\alpha }_{n\varphi }$ approaches zero in $-3{\rm{c}}{\rm{m}}\le r-a\le -0.3{\rm{c}}{\rm{m}}$, while outside the region ${\alpha }_{n\varphi }$ becomes negative. Non-adiabatic response of electrons on potential perturbation that gives birth to the outward particle flux is weakened. Figure 3(h) shows profile of the particle flux normalized by mean density. The value in L-mode is in a similar order to that reported in Ref.^32^ in which the turbulence particle flux is identified as the dominant loss channel of plasma density. As a result of changes mainly in the density fluctuation amplitude and the cross phase, the particle flux is drastically reduced.

**Figure 4:**
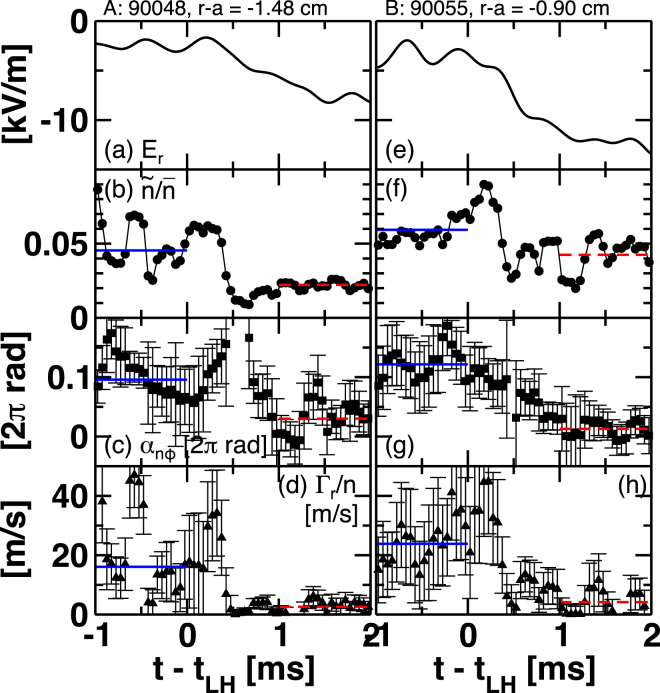
Time traces of (**a**) radial electric field ${E}_{r}$, (**b**) relative density fluctuation amplitude, (**c**) cross phase between density fluctuation and potential fluctuation, and (**d**) particle flux at $r-a=-1.48$ cm. (**e**–**h**) Those at $r-a=-0.90$ cm. Blue-solid and red-dashed horizontal lines in (**b**–**d**) and (**f–h**) are mean values in L-mode and MH-mode, respectively.

Detailed time evolution during the transition is shown in Fig. 4. Left and right columns are time traces for $r-a\sim -1.48$ cm (#90048) and for $r-a\sim -0.90$ cm (#90055), respectively, in which either *Z*_1_ or *Z*_2_ predominantly varies. Time scale of change in *E*_*r*_ non-uniformity is considered to be equivalent as that in *E*_*r*_. For both cases, time scale of the ${E}_{r}$ transition is the order of 100 *μ*s. After the transition, a slow draft of *E*_*r*_ further deepens the transport barrier in the MH-mode state. The relative density fluctuation amplitude immediately responds to change in *E*_*r*_ with the time scale of the order of 100 *μ*s. The change in the amplitude saturates prior to *E*_*r*_. Meanwhile, the change in the cross phase is much slower. The time scale of the change is 500 *μ*s to 1 ms. These tendencies are common for both cases. The difference of time scale may suggest different underlying physics for density fluctuation amplitude suppression and cross phase modification. Note that the potential fluctuation amplitude is less sensitive to the change in the radial electric field in particular at the *E*_*r*_-well location as shown in Fig. 3. Models taking into account the different responses in density fluctuation and potential fluctuation are highly desirable in future.

**Figure 5:**
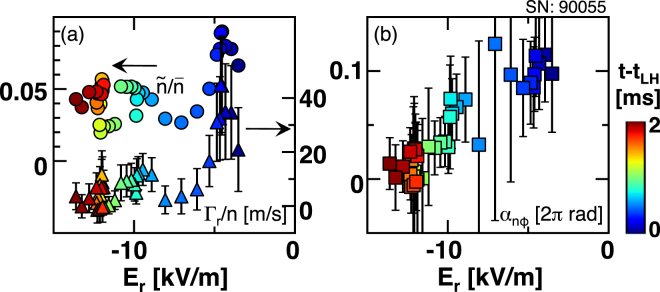
(**a**) Relative density fluctuation amplitude and particle flux and (**b**) cross phase plotted as a function of radial electric field ${E}_{r}$. Color of symbols corresponds to time, which is shown by the color bar.

For the case of #90055, the relative density fluctuation amplitude, the cross phase, and the particle flux are plotted as a function of *E*_*r*_ in Fig. 5. The relative density fluctuation amplitude decreases as *E*_*r*_ grows when ${E}_{r} > -7$ kV/m. Correspondingly, the particle flux decreases, suggesting that the paradigm of turbulence transport suppression through amplitude effectively works when the *E*_*r*_ non-uniformity is relatively small. Throughout the change in *E*_*r*_ the cross phase gradually approaches zero. With larger value of the *E*_*r*_ non-uniformity the change in the cross phase dominates change in the particle flux.

## Discussion and summary

Even inside radii where radial electric field non-uniformity are small, turbulence amplitude in both relative density fluctuation and potential fluctuation are reduced, as observed in CCT^18^. One of the possible mechanisms that can bring this disparate space turbulence suppression is “*turbulence spreading*”^33^. Dynamic inward transmission of a turbulence packet was observed in a limit cycle oscillation event^26^. This observation can provide a clue to address a long-standing mystery, that is, the fast improvement of core confinement by edge transport barrier^34^.

In conclusion, we investigated response of turbulent particle flux on radial electric field shear and curvature measured by heavy ion beam probe. Particle flux was mainly suppressed by reducing density fluctuation amplitude and cross phase between density fluctuation and potential fluctuation. Both radial electric field shear and curvature were responsible for transport suppression. Turbulence amplitude reduction immediately responded to the growth of the radial electric field non-uniformity and saturates, while cross phase continuously approached zero.

## Methods

### JFT-2M

The experiments were conducted in the JFT-2M tokamak, which has a major radius of 1.3 m and a minor radius of 0.3 m. Plasma was auxiliary heated by a co-injected neutral beam (NB) with the power of 750 kW, which is just above the L-H transition threshold. Line averaged electron density was $1.1\times {10}^{19}\,{m}^{-3}$ before the L-H transition. An upper single-null divertor configuration was employed with the $\nabla B$ drift of ion directed toward the X-point. Other operation parameters were as follows: toroidal magnetic field at the magnetic axis ${B}_{{\rm{t}}}$ of 1.17 T, safety factor at the flux surface enclosing 95% of the total poloidal flux, ${q}_{95}$, of 2.9, and plasma current *I*_p_ of 190 kA.

### Heavy Ion Beam Probe (HIBP)

In order to diagnose electrostatic potential *ϕ*, heavy ion beam is injected from the top-side of the torus, which is ionized doubly inside the confined plasma. By analyzing the secondary beam energy, *ϕ* is given at four sample volumes ($6\,{\rm{mm}}\times 2\,{\rm{mm}}$) with a sampling rate of 1 *μ*s^30^. Radial distance between each sample volume projected in the outer mid-plane is ~2.5 mm. With precise tuning of the HIBP measurement conditions, such as the primary beam energy, the toroidal magnetic field, and the incident angle of the beam, measurement positions can be scanned in an edge region ($-5\,{\rm{cm}} < r-a < 0\,{\rm{cm}}$) on a shot-to-shot basis. Relative secondary beam current fluctuation ${\tilde{I}}_{{\rm{HIBP}}}/{\bar{I}}_{{\rm{HIBP}}}$ is regarded to be equivalent to relative electron density fluctuation $\tilde{n}/\bar{n}$, since beam attenuation effect is estimated to be negligibly small at the edge^35^. Gradient of quantities is defined by the finite difference of the neighboring sampling volumes.

### Turbulence suppression model

In the newly developed theoretical model^12^, amplitude of turbulence having a perpendicular wavenumber *k* is reduced by the shear factor *Z*_1_ and the curvature factor *Z*_2_ as $I/{I}_{0}={\mathrm{[1}+{(k{\rho }_{{\rm{i}}})}^{-2}({Z}_{1}+{Z}_{2})]}^{-1}$, where *I* and *I*_0_ are the reduced turbulence amplitude and the intrinsic turbulence amplitude without *E*_*r*_ effects, respectively. Shear factor ${Z}_{1}\equiv {\rho }_{{\rm{i}}}^{2}{({V}_{{\rm{d}}}B)}^{-2}{E}_{r}^{^{\prime} 2}$ and curvature factor ${Z}_{2}\equiv -{\rho }_{{\rm{i}}}^{2}{({V}_{{\rm{d}}}B)}^{-2}({E}_{r}-{V}_{{\rm{t}}{\rm{o}}{\rm{r}}}{B}_{\theta }){E}_{r}^{^{\prime\prime} }$ are defined as indicators of significance of shear decorrelation and of modulational coupling, respectively, where ${\rho }_{{\rm{i}}}$ is the ion gyro-radius, ${V}_{{\rm{d}}}\equiv T|n^{\prime} |/enB$ is the diamagnetic velocity, and prime is the radial derivative. In the present case, ${E}_{r}\gg {V}_{{\rm{tor}}}{B}_{\theta }$ is considered to hold^7^ so that the toroidal velocity correction term for *Z*_2_ is neglected. Recent investigation in JT-60U^8^ showed an important role of not only *Z*_1_ but also of *Z*_2_ by referring to temperature gradient profile as an indicator of confinement intensity.
